# Supplementary material for: Are carbon emissions trading and green financial instruments synergistic? -Comprehensive quantitative research based on content analysis
Source: PLoS One. 2024 Mar 7;19(3):e0298601. doi: 10.1371/journal.pone.0298601 (PMC10919601; doi:10.1371/journal.pone.0298601)
Supplement: S1 Data — (ZIP) [file pone.0298601.s001.zip › Data/地级市Z_F工作报告.zip!/║■▒▒/╦μ╓▌╩╨/└┤╫╘╣1⁄2╓┌║┼í╛┬φ┐╦╩2╛▌═°í┐_2016.docx]

各位代表：

现在，我代表市政府向大会报告工作，请予审议，并请各位政协委员和列席人员提出意见。

一、“十二五”时期经济社会发展回顾

“十二五”以来，在省委、省政府和市委的坚强领导下，全市上下深入贯彻落实科学发展观和党的十八大精神，解放思想，攻坚克难，积极应对复杂多变的外部环境，努力缓解经济下行的巨大压力，全市经济社会保持快速健康发展的良好态势，“十二五”发展的主要目标任务顺利完成。

过去五年，战略定位开启新格局。“圣地车都”战略上升为省级战略，围绕《随州“圣地车都”战略发展规划》出台实施了专汽、冶金、建材、旅游等产业中长期发展规划，同时，我市获批创建湖北省可持续发展实验区，并被全域纳入大别山革命老区振兴发展、长江经济带、汉江生态经济带等战略规划。一系列重大战略的部署实施，为我市进一步明确了战略方位，细化了战略目标，廓清了战略路径，引领并推动着全市经济社会不断向前发展。

过去五年，综合实力跨上新台阶。“十二五”时期，全市主要经济指标增速持续好于优于全国、全省平均水平，在全省实现“保五争三”，经济运行质量和效益不断提升。地区生产总值实现翻番，达到785亿元，年均增长11.2%，实现规模工业增加值362亿元、规上固定资产投资907.7亿元、社会消费品零售总额399.6亿元、地方公共财政预算收入43.3亿元，年均分别增长15.1%、31.7%、15%和35.4%，节能减排任务全面完成，万元生产总值能耗下降13%。

过去五年，产业发展迈出新步伐。转型升级不断加快，产业结构出现积极变化，三次产业之比由 “十一五”末的21.6：45.2:33.2调整为17:49:34。农业基础夯实加固，实现粮食增产、农业增效、农民增收，农业总产值达到239亿元，是2010年的1.6倍。工业经济难中求进，以汽车机械、食品工业、冶金建材、医药化工、电子信息、战略性新兴产业等六大产业为支撑的工业体系基本形成，实现工业总产值1335亿元，是2010年的2.6倍，特别是新能源产业异军突起，风电、光伏发电装机容量达到60万千瓦，我市一跃成为全省新能源第一大市。第三产业蓬勃兴旺，文化旅游健步发展，连续七年成功举办寻根节，炎帝故里、大洪山、千年银杏谷、西游记公园等一批重点景区全面建成，接待游客人数、旅游综合收入年均分别增长24%和23%，金融保险、信息服务、快递物流等现代服务业加速发展。

过去五年，城乡面貌发生新变化。围绕“双百”城市建设，坚持规划引领、基础先行、建管并重，全市常住人口城镇化率达到47.9%。城市建设稳步推进，文化公园、滨湖体育运动场、青年路大桥等重点市政项目相继建成使用，城南、城东和城北新区建设提速，中心城区建成区面积达到70平方公里，随县新县城基本建成，广水“一河两岸”初具规模，一批特色镇、重点镇加快发展，“一主两翼、多点支撑”的城镇化格局逐步形成。城市名片越擦越亮，成功创建国家森林城市、国家园林城市、全国绿化模范城市和湖北省省级文明城市、首个环保模范城市，省级卫生城市顺利通过复查验收，随州的知名度、美誉度进一步提升。

过去五年，改革开放实现新突破。新一轮政府机构改革全面铺开，文体广电、医疗卫生、财税金融、食药监管、城管综合执法等重点领域改革顺利推进。行政审批制度改革深入实施，审批流程得到再造，审批效率进一步提升。投融资体制改革取得成效，融资渠道向多途径转变，裕国菇业、双剑风机、广彩印刷等3家企业挂牌“新三板”，10家企业挂牌“四板”，城投、建投债券成功发行并募集资金32亿元。对外开放进一步扩大，累计出口54亿美元，香菇出口稳居全国地级市首位、农产品出口稳居全省第一、机电产品出口快速增长，实际利用内联资金673.4亿元、利用外资4.1亿美元，引进世界500强企业及央企省企39家，混合所有制企业达到27家。

过去五年，人民生活有了新改善。城镇居民人均可支配收入22761元、农村居民人均可支配收入13093元，年均分别增长11.2%、13.1%。社会保障体系逐步完善，社会保险、住房公积金覆盖面不断扩大，城乡居民养老保险参保率100%。就业形势稳定，城镇登记失业率控制在4.5%以内。实施“三民”工程、城乡网格化管理、“三调联动”等，进一步创新和加强社会治理。教育、文化、卫生、体育等社会事业取得长足发展，市政府承诺的实事项目全面完成，住房难、上学难、看病难、出行难等问题得到有效缓解。

与此同时，新闻出版、文联、社科联、外事侨务、对台、民族宗教、对口援疆和国防动员工作收获新成果，工会、青年、妇女儿童、残疾人、红十字会、无偿献血、老龄和慈善事业呈现新气象，审计、统计、国调、质监、国资监管、物价、供销、机关事务、公共资源交易管理、档案、保密和地方志工作有了新提升，邮政、通讯、无线电、石油石化、烟草专卖、民防、消防、水文和气象等工作取得新业绩。

特别是在过去的一年，面对经济下行的重重困难，我们坚持竞进提质、升级增效、先行先试、特色发展，统筹推进稳增长、促改革、调结构、惠民生、防风险各项工作，巩固和扩大了全市经济社会发展的积极态势，较好地完成了市三届人大五次会议确定的各项目标，实现了“十二五”圆满收官。2015年地区生产总值同比增长8.8%左右，规模工业增加值增长9.3%、居全省第4位，规上固定资产投资增长18.1%、居全省第4位，地方公共财政预算收入增长18.2%、居全省第4位，社会消费品零售总额增长12.8%、居全省第5位，实际利用外资增长14.5%，城镇居民人均可支配收入增长8.6%，农村居民人均可支配收入增长9.3%，我市主要经济指标增速在全省实现“保五争三”目标。

（一）锐意推进改革，发展动力更加强劲。政府职能加快转变，事业单位分类改革有序推进，议事协调机构清理精减率达到92%。行政审批提速提效，锁定265个审批事项，大力推行网上审批、并联审批，17个市直部门投资项目联合审批平台试运行，“三证合一、一照一码”商事制度改革在全省率先实施。创新财税体制，“营改增”逐步推进，强化预算管理，统筹财政专项资金1.8亿元，盘活财政存量资金10.4亿元，资金使用效益显著提升。产权制度改革实现突破，不动产统一登记试点工作在全国先行先试，并取得成功经验，农村土地承包经营权确权登记颁证工作在全省率先完成。多层次资本市场建设破冰前行，在全省率先设立1.25亿元中小企业续贷周转金，为企业解决12亿元“过桥”资金，政银集合贷、助保贷帮助117家企业贷款11.3亿元，全市新增信贷投放近80亿元，新增“新三板”挂牌企业2家、“四板”挂牌企业4家，裕国菇业从资本市场实现直接融资1.1亿元。深入推进混合所有制改革，新增混合所有制企业6家，同星农业与正大集团成功重组。创新驱动成效显著，实现高新技术产业增加值80亿元，占地区生产总值比重达到10%以上，湖北省专汽研究院等3家单位获批博士后科研工作站，随州高新区成功升级为国家高新区。

（二）着力调优结构，产业体系更加健全。农业基础持续稳固。粮食增产2.5万吨、总产达到163.4万吨，食用菌、马铃薯等优势农产品种植规模不断扩大。农业产业化进程加快，新增省级农业产业化龙头企业9家，新登记农民合作社391家、家庭农场96家，新增土地流转16.5万亩。农村基础设施不断改善，争取土地整治项目10万亩，除险加固病险水库161座。工业经济加快转型。新进规模企业101家，亿元企业突破400家，成功创建国家新型工业化产业示范基地、国家首批应急产业（专用车）示范基地和“宽带中国”示范城市。六大产业板块不断壮大，占工业总产值比重达到93.8%。专汽产业回暖上扬，专用车产量首次突破10万辆、增长15%以上，新增专汽资质企业3家，成功举办2015年中国·随州专汽发展论坛，被授予国家专用汽车质量提升示范区、国家级出口专用车产业质量安全示范区；食品工业稳步增长，实现产值323亿元，增长12.5%；新能源等战略性新兴产业加速扩张，电子信息进入省重点产业集群。第三产业迅猛发展。文化旅游亮点纷呈，乙未年寻根节、第25届中国（随州）兰博会、大洪山慈恩寺本焕长老舍利塔安放系列活动、2015中国·随县香菇节等成功举办，九口堰新五师纪念园建成开园并获批全国爱国主义教育基地，全年共接待游客1760万人次、旅游综合收入109.5亿元，同比分别增长11%、12%，其中大洪山风景区接待游客人数增幅超过40%，西游记公园、西游记漂流和千年银杏谷接待游客人数首次突破百万。电子商务不断拓展，高新区电商产业园入驻企业50多家，淘宝·特色中国随州馆入驻企业200多家，苏宁云商随州馆开馆上线，广水市、随县分别被命名为国家级、省级电子商务进农村示范县市。

（三）紧盯项目建设，投资拉动更加有力。

131个省市重点项目开工，其中12个省级项目完成投资49亿元。一批重大项目顺利推进，汉十铁路、鄂北水资源配置工程和华能随州燃煤电厂全面开工、以500千伏输变电站为主体的区域电网新建和农网改造加快实施、全省最大的马鞍山100兆瓦地面光伏电站建成运营、东风车轮1000万套车轮生产线正式投产。创新政府和社会资本合作模式，城南污水处理厂等PPP项目进展顺利。招商引资取得新进展，新引进固定资产投资亿元以上项目40个，争取中央和省预算内项目资金8.6亿元、中央专项建设基金15.9亿元，实际利用内联资金240亿元、同比增长22%。结合“十三五”规划编制，新策划2351个亿元以上重点项目，其中247个纳入国家重大工程、省重大专项项目库。

（四）坚持建管并举，城镇功能更加完善。新一轮“五城同创”有序进行，城市建设和管理水平逐步提升。一批重点基础设施项目加快推进，东外环公路全线贯通，博物馆扩建、淅河大桥改造、季梁大道延伸段等已经完工，规划展览馆布展开放，传媒大厦、解放西路、神农大道淅河段、高新区“一河两岸”改造等抓紧建设。城市管理综合执法能力进一步提升，数字城管指挥中心试运行，地下管网普查工作进展顺利。开展“绿满随州”行动，完成造林27.8万亩，超过省定目标25%，中心城区新增绿地61.8万平方米。“美丽乡村”建设成果丰硕，2个镇获国家生态镇、2个镇获省“楚天杯”、10个村成为省宜居村庄，长岗镇被命名为全国特色景观旅游名镇、省级旅游名镇，我市农村生活垃圾治理工作居全省第一方阵。

（五）高度关注民生，人民福祉更加增进。财政用于民生领域的支出达到103亿元，占公共财政预算支出的79%。全市新增就业2.2万人，新增小额担保贷款1.2亿元，扶持创业1113人，新登记市场主体3.3万户。社会保险覆盖面不断扩大，城乡居民养老保险参保103.3万人，医疗、失业、工伤、生育保险实现市级统筹。公立医院分级诊疗体系加快建立，县级公立医院综合改革实现全覆盖，新农合参合率稳定在99%以上，大病商业保险为1.2万参保人员支付保险3600多万元。持续推进“两室联建、医养融合”，建成社区居家养老服务中心136个和农村互助式照料活动中心259个。学前教育资源较快增长，义务教育办学条件不断改善，投入1.2亿元改扩建教学及辅助用房、生活用房、运动场，高新区季梁学校建成开学，城南新区白云湖学校建成交付，教育质量进一步提高，高考实现新突破。保障性住房、棚户区改造住房开工9697套，基本建成6534套，分配入住5258套。住房公积金新增缴存4500人，新增归集5.4亿元。加大环保执法力度，切实开展大气污染专项整治行动，PM10均值比上年下降4.63%，空气质量综合指数排名全省第4；城区饮用水源地综合治理纵深推进，封江供水复线工程全面完成，水王福窑取水口正式关闭，中心城区生活污水集中处理率达到90%，新解决23.4万人农村安全饮水问题。深入实施食品药品安全示范创建和专项整治行动，努力保障食品药品安全。全面打响扶贫攻坚战，因村因户开展精准扶贫，全市扶贫小额贷款规模达到5000万元，实现脱贫2万人。完成通村公路450公里，“村村通客车”实现全覆盖。加快建设“平安随州”，以信息技术和“7+N”常态化巡防机制为支撑的立体化防控体系不断完善。大力开展“双拥”创建，军地融合深入推进。高度重视安全生产工作，全年未发生一起较大以上安全生产事故。着力化解各类矛盾纠纷和信访积案，社会大局和谐稳定。

（六）不断转变作风，自身建设更加扎实。自觉接受人大法律监督和政协民主监督，全年办理人大议案建议、政协建议案提案276件，满意率进一步提升。持续推进法治政府建设，“六五”普法依法治理工作顺利迎检，普遍建立法律顾问制度逐步实施。坚持开展 “周报月评年考”，深入开展“乡镇大比武”，全面开展“三严三实”专题教育活动，扎实开展“四项专项整治、三个专项治理”，大力整治“不作为、慢作为”现象，驰而不息纠正“四风”，不断推进“两个责任”落地生根。认真落实省委巡视反馈意见，积极进行市级预算执行和其他财政收支审计整改，着力构筑风清气正的政治生态，努力打造阳光政府、廉洁政府。

各位代表！“十二五”时期随州经济社会发展取得的成就，是省委、省政府正确领导的结果，是市委科学决策的结果，是市人大加强监督、市政协积极参政议政的结果，是全市广大干部群众和社会各界团结奋斗的结果。在此，我代表市政府，向全市人民，向各位代表、政协委员，向各民主党派、工商联、人民团体和无党派人士，向驻随部队、武警官兵和政法公安干警，向所有关心、支持和参与随州经济社会建设的同志们、朋友们，表示衷心的感谢，致以崇高的敬意！

回顾“十二五”期间的工作，我们清醒地认识到，当前我市经济社会发展还存在一些不容忽视的困难和问题：一是保持经济持续快速增长压力加大。受多方面因素特别是宏观经济下行的影响，我市部分经济指标虽保持增长，但增速下滑；部分企业经营较为困难，要素制约突出，特别是融资渠道不畅，盈利空间变小。二是产业结构仍需深入调整。产业实力整体较弱，层次不高，竞争力不强，创新能力不足，传统产业占比较大，高新技术产业和第三产业比重偏低。三是民生改善任务依然繁重。城乡基础设施建设仍有欠账，社会事业发展相对不足，公共服务能力和水平尚需提升，广大群众反映比较集中的一些热点难点问题还没有得到很好解决。四是行政效能有待进一步提高。少数部门和工作人员依法行政观念和为民服务意识较差、能力不足、作风不优，遇事推诿、办事拖拉、为官不为等现象仍然不同程度存在。对此，我们一定高度重视，积极采取措施，认真加以解决，努力取得人民群众满意的效果。

二、“十三五”时期经济社会发展的主要思路

“十三五”时期是全面建成小康社会的决胜阶段，需要我们切实把思想和行动统一到中央、省委省政府和市委的决策部署上来，科学认识和准确把握经济新常态，进一步增强跨越发展的机遇意识、责任意识和忧患意识，充分利用各种有利条件，加快解决突出矛盾和问题，全力以赴推进经济建设、政治建设、文化建设、社会建设和生态文明建设。

“十三五”时期全市经济社会发展的指导思想是：高举中国特色社会主义伟大旗帜，全面贯彻党的十八大和十八届三中、四中、五中全会精神，深入落实习近平总书记系列重要讲话精神，以“四个全面”战略布局为统领，以“五大发展理念”为引领，认真践行“三维纲要”，实施“圣地车都”战略，按照“竞进提质、升级增效，以质为帅、量质兼取”的工作方针，提升特色、放大优势、壮大实力，加快建设鄂北门户城市、区域性中心城市，确保与全省同步全面建成小康社会。

主要目标是：力争地区生产总值在“十二五”末基础上翻一番，达到1400亿元，年均增长9%；规模工业增加值年均增长9%；全社会固定资产投资年均增长15%；社会消费品零售总额年均增长11%；外贸出口总额年均增长8%；地方公共财政预算收入年均增长11%；城乡居民人均可支配收入年均增长9%。

“十三五”时期，我们将努力在以下五个方面实现突破：

（一）在做大产业总量和提高效益上实现重大突破。积极引导产业合理布局，大力优化三次产业结构，促进产业融合发展，着力构建布局科学、分工合理、特色鲜明的现代产业体系。一是巩固发展现代农业。加快推动农业发展方式转变，建立完善现代农业生产体系、经营体系、质量安全全过程监管体系、科技创新推广体系、社会化服务体系。推进优势特色农产品区域化布局、规模化建设、标准化生产、产业化经营，大力提高农业综合生产能力、抗风险能力和市场竞争能力，确保粮食安全和农产品有效供给，实现传统农业大市向现代农业强市转变，全面建成国家现代农业示范区。二是提升工业整体竞争力。对接“中国制造2025”，推进工业化和信息化融合发展，改造提升传统产业，大力发展先进制造业，培育壮大战略性新兴产业，促进专用汽车及零部件、风机、香菇、铸造、电子信息等五个省级重点成长型产业集群持续健康发展，力争建成专用汽车、食品工业两大“千亿元产业”，成功创建国家新能源示范城市。三是加快发展现代服务业。坚持生产性服务业与生活性服务业并举，着力培育消费新热点、新业态，创造新供给，释放新需求，增添新动力，推动服务业大发展，力争服务业增加值占GDP比重达到37%。实施文化旅游产业支柱化培育工程，加强品牌营销，提升服务水平，着力打造全国知名的旅游目的地、集散地。

（二）在改革创新和扩大对外开放上实现重大突破。抓住制约科学发展的关键症结，通过改革破解发展难题，依靠创新厚植发展优势，立足开放激发发展活力。一是深化更有效的改革。围绕事关全局的重要领域和关键环节，切实加快农业农村、行政管理、财税金融、社会发展等体制机制改革步伐。强化市场主体地位，推进要素配置市场化改革，努力消除民间资本进入的隐形壁垒。二是实施更主动的开放。大力实施开放先导战略，坚持对内对外双向开放，立足“引进来”，多途径扩大招商引资，提升利用外资水平，着眼“走出去”，多渠道开辟出口市场，推动外贸优化升级，加快提升开放型经济发展水平。三是加强更紧密的合作。积极融入国家、省重大区域战略，探索区域协同发展机制，不断拓展区域发展空间，重点加快大洪山旅游资源整合，推进大别山区域合作示范区和国家承接产业转移示范区建设。四是开展更广泛的创新。围绕创新型城市建设，不断推进科技、制度、产业、要素等各方面创新，推动跨领域跨行业协同创新，让创新在全社会蔚然成风，积极培育发展新动力。

（三）在城镇发展和生态文明建设上实现重大突破。坚持“五个统筹”，走新型城镇化发展道路，着力构建“一主两翼、多点支撑”城镇化格局，力争常住人口城镇化率达到56.6%。一是不断提升城市功能。紧盯“双百”城市目标，坚持“东进、西优、南拓、北调”，不断拓宽城市骨架。统筹推进老城区和城南、城东、城北新区建设，重点推进城南高铁片区建设。全面提升基础设施水平，大力完善城市功能，进一步增强产业支撑能力、公共服务能力和城市综合承载力。二是不断推进城乡发展。实施新型城镇化和新农村建设“双轮”驱动，建立城乡融合的体制机制，形成以工促农、以城带乡、工农互惠、城乡一体的新型工农城乡关系。提升随县、广水城市功能，坚持“一镇一特色”建设新型小城镇，坚持“一村一品牌”建设美丽宜居新农村。三是不断加强生态文明建设。坚持生态立市和绿色发展理念，以提高环境质量为核心，实行最严格的环境保护制度、水资源管理制度和节约用地制度，促进资源科学开发与高效利用，大力发展低碳循环经济，促使单位GDP能耗、水耗、二氧化碳排放和主要污染物排放大幅下降，努力建设国家生态文明先行示范区。

（四）在民生改善和社会事业发展上实现重大突破。继续加大社会事业支出，推进基本公共服务均等化，确保发展成果由人民共享。一是抓好社会就业创业。积极拓展就业渠道，实现更加充分的就业，确保5年新增就业6万人。推进创业型城市创建，积极实施众创、众包、众扶、众筹，力争创建省级创业示范基地8个以上。二是抓好社会保障工作。推动社会保险从制度全覆盖到人员全覆盖，加快构建社会保险、住房保障、社会救助、福利事业相互衔接的社会保障体系。三是抓好社会事业发展。坚持教育优先发展，不断改善办学条件，切实提高教育质量。大力弘扬特色传统文化，完善城乡公共文化服务体系，积极发展文化体育事业。围绕健康随州建设，加快完善新型城乡医疗卫生服务体系，着力构建食品药品安全质量源头可溯、全程可控、风险可防、责任可究、公众可查的全过程安全监管机制。四是抓好社会治理创新。加强社会治理能力建设，深入推进“三民”工程，探索完善新形势下的安全生产、综治维稳、防灾减灾、社会组织建设等工作新机制。

（五）在精准脱贫和小康随州建设上实现重大突破。围绕全面建成小康社会的总目标，突出推进精准扶贫，在2018年底前实现14.6万贫困人口全部脱贫、126个贫困村全部“出列”。一是实施产业扶贫。积极探索符合贫困地区实际且与贫困户利益相关联的产业扶贫模式，发挥旅游开发、光伏发电的产业扶贫作用，确保贫困户合理分享资源开发效益。二是促进自主脱贫。深入实施贫困劳动力“雨露计划”，大力推进职业技能、实用技术和致富带头人创业培训，全面加强贫困劳动力能力建设，促进就地转移就业、创业稳定增收。三是推进整村扶贫。加大贫困村交通、电力、水利等基础设施和文化体育设施建设力度，分期分批分类对贫困村实行环境综合治理。四是创新扶贫机制。以市县两级建立“七联”工作队为抓手，建立健全驻村帮扶机制，完善部门联村、干部联户全覆盖帮联责任制。对各类涉农资金“打捆”使用，综合运用低保等各种兜底政策，推进金融扶贫，鼓励动员社会力量参与扶贫开发。

三、努力做好2016年各项工作，为“十三五”开好局

2016年是“十三五”的开局之年，也是全面建成小康社会的关键之年，做好今年的工作至关重要。当前，我们既拥有难得的机遇，也面临严峻的挑战。从机遇看，世界经济继续缓慢复苏，我国经济长期向好的基本面没有改变；我市被纳入大别山革命老区振兴发展规划、长江经济带、汉江生态经济带等一系列国家级、省级战略，为我市扩大对外开放、加强区域合作、承接产业转移提供了广阔空间；经过“十二五”的发展，我市资源、区位和产业优势正在扩大，一批带动性强、效益较好的重大项目相继动工建设，经济发展具有总量扩张和产业升级的强大内生动力。从挑战看，“三期叠加”阵痛加深，多重困难相互交织，经济下行压力加大，我们既要保持经济平稳较快增长，又要调结构、促转型，还要保民生、防风险，任务十分艰巨。机遇与挑战并存，要求我们必须保持清醒的头脑，在科学把握形势的基础上，努力抢抓机遇，积极应对挑战，以更强的信心、更大的作为、更多的担当，来推动经济社会更好更快发展，为“十三五”开好局、起好步。

今年全市经济社会发展的主要预期目标是：地区生产总值同比增长9%左右，规模工业增加值增长9%，全社会固定资产投资增长16.5%，社会消费品零售总额增长12.5%，外贸出口总额增长10%左右，实际利用外资增长10%，地方公共财政预算收入增长11%，城镇、农村居民人均可支配收入分别增长8.5%和10%左右，主要污染物总量减排、环境质量控制等全面完成省考核目标。

围绕上述目标，根据中央经济工作会议和省委十届七次全会暨全省经济工作会议、城市工作会议精神，今年市政府将重点抓好以下八个方面的工作：

（一）全力深化重点领域改革。大力发扬“工匠精神”，扭住关键，精准发力，着力解决“最后一公里”问题，确保各项改革落到实处、见到实效。

1、加快职能转变深化改革。深入推进政府机构改革，进一步转变政府职能。继续推进行政审批制度改革，巩固和扩大商事制度改革成果，着力优化行政审批流程，降低行政审批收费，提高行政审批效率。加快事业单位分类改革，做好人事制度、收入分配、社会保险制度等配套改革。全面推进公务用车和机关事业单位养老保险改革。深化司法体制改革，进一步促进司法公正。

2、加强公共服务供给深化改革。围绕社会关切、群众关心的重要领域关键环节加大改革力度，努力破解一批制约发展、影响民生的热点难点问题。推进教育领域改革，办好基础教育综合改革试验区。推进文化体制改革，理顺文化管理体制，增强文化事业发展活力，推动文化产业转型升级，扩大和引导文化消费。推进医疗卫生体制改革，深化公立医院综合改革，全面推动“医疗、医药、医保”联动，加快建立分级诊疗、双向转诊制度，继续做好“两室联建、医养融合”工作。

3、优化资源配置深化改革。加快财税改革。坚持开源与节流并重、增收与管理并举，完善政府预算体系，切实盘活财政存量资金，不断提升配置效益；深化税收征管体制改革，推进税收现代化建设。加快金融改革。创新投融资渠道，积极参与长江经济带产业基金，推动 “区域集优”融资模式实现突破，力争高新区高新投、建投债券二期、玉龙公司企业债券成功发行，积极参股省再担保集团；加强中小企业担保体系建设，切实用好、做大中小企业续贷周转金，推广助保贷、政银集合贷、出口退税贷等金融产品模式，扩大融资规模，降低融资成本，努力破解融资瓶颈；支持企业上市，力争泰晶科技成功上市，天星粮油、双星生物等更多企业在“新三板”、“四板”挂牌，不断推进资产证券化。加快农村产权制度改革。规范农村土地承包经营权流转，探索“两权”抵押新模式，努力激活农村资源要素。

（二）全力抓好项目投资工作。坚持把投资和项目作为稳增长的主抓手，千方百计扩大有效投资，进一步做大发展底盘。

1、抓谋划、强储备。根据中央及省级预算内投资方向和国家专项建设基金投资重点，谋划储备一批重大基础设施建设、创新和结构调整、社会事业和社会治理、节能环保与生态建设等方面的项目，充实完善“十三五”项目库，编制实施三年滚动投资计划，切实对接国家“11个工程包”、20个省级投资专项等，争取更多项目纳入国家和省投资计划。

2、抓拓展、挖潜力。紧盯上级政策动向、财政资金流向和社会资本风向，加大对上衔接、争取力度，确保我市争取中央、省补助投资份额稳中有升。鼓励和引导民间投资，更好发挥民间投资的主力军作用，放宽民间资本市场准入，优化民间投资项目库，加大PPP项目推进力度，不断扩展投资渠道、扩大投资规模。

3、抓建设、增后劲。深化“项目建设大行动”，按照“签约项目抓落地、落地项目抓开工、开工项目抓进度、竣工项目抓投产”的要求，强化项目调度，制定项目推进路线图，落实现场观摩、督办通报和绩效考核等制度，加快推进汉十铁路、鄂北水资源配置工程、华能随州燃煤电厂、总装机40万千瓦的风力和光伏发电、青啤公司40万千升啤酒生产线、健民叶开泰国药、红星美凯龙国际广场等重大项目建设，确保投资持续稳定增长。

（三）全力优化调整产业结构。认真落实去产能、去库存、去杠杆、降成本、补短板“五大任务”，协同发展一二三产业，不断构建现代产业体系。

1、坚持固本强基，着力推进农业现代化。加快农业结构调整，不断提高农业综合实力。稳定粮食生产，确保全年粮食产量达到165万吨以上。调整优化种养业结构，壮大食用菌、马铃薯、优质桃、蔬菜、茶叶、木本油料等特色种植板块，提升畜禽、水产养殖发展规模和水平。加大农技推广和服务力度，普及测土配方施肥技术，推广轻简栽培技术。规范土地流转，大力培育种养大户和家庭农场，发展农民合作社，壮大农业产业化龙头企业，加快构建新型农业经营体系。继续实施淮河源国家湿地公园、蕙兰谷生态园、丰年国际核桃园等 “十大林业生态示范工程”，推动林业经济发展。

2、坚持做大做强，着力推进新型工业化。突出发展先进制造业，深化“两化”融合，以“智造”为主攻方向，切实加快转型升级步伐。围绕主导产业，壮大整体实力。进一步提高专汽及零部件行业发展水平，抓好国家应急产业（专用车）示范基地和国家专用汽车质量提升示范区建设，推动产品向信息化、智能化、轻量化发力；进一步做好食品工业，推进农产品精深加工，力争农产品加工业产值与农业总产值之比达到2.3：1；进一步改造提升冶金建材、医药化工业，加速发展新能源、电子信息等战略性新兴产业。大力发展混合经济，推动民营、国有、外资等多种经济形式融合发展，不断提升与央企省企的对接合作质效。坚持有保有压、有扶有控，做好“加减乘除”法，推进市场主体提质增效、优胜劣汰。深入实施“中小企业成长工程”，力争新进规模企业60家以上。继续落实领导包保重点企业制度，不断完善“一企一策”、“大企业直通车”和“绿色通道”服务，大力开展“万名干部进万企”活动，出实招帮扶企业，助力实体经济行稳致远。

3、坚持消费引领，着力推进现代服务业。顺应消费对经济增长拉动作用持续增强的大势，继续以特色旅游、金融保险、现代物流、信息服务、养老健康、社区服务等行业为重点，全面提升服务业发展水平，引导消费观念转变，发展新的消费模式，大力培育新的经济增长点。加快发展文化旅游，实施“智慧旅游”工程，推动旅游与相关产业融合，抓好重点景区景点建设，精心打造一批特色文化旅游项目，全力办好丙申年寻根节，切实推动炎帝故里文化旅游区5A级景区创建取得突破，有序推进大洪山风景区5A级景区创建，确保新增4A级景区1个以上，力争接待游客人次达到2000万，实现旅游综合收入120亿元。加快县域金融工程建设，新引进一家股份制商业银行，力争新增信贷投放100亿元。加快发展现代物流，积极打造物流平台，提高综合效益。加快发展电子商务，建设一批专业性电子商务平台，全面推进 “互联网+”行动，积极推动移动互联网、云计算、大数据、物联网等与经济社会融合发展。

4、坚持协调发展，着力推进县域经济。充分发挥县域经济统筹工业化、新型城镇化和农业现代化的重要载体作用，引导资金、项目、技术、人才等要素向县域流动，进一步培育壮大随县农产品加工、广水风机和智能装备制造、曾都专汽及零部件等特色产业集群，增强县域经济实力。充分发挥国家高新区辐射带动作用，进一步加强各级各类开发区建设，优化功能布局、产业定位、管理机制，促使产业向园区聚集、企业向园区靠拢，推动各开发区向特色化、集约化、生态化方向发展，打造经济建设的主战场、主阵地。

5、坚持科技创新，着力推进创新驱动。以科技创新为核心，大力实施创新驱动战略，不断增强经济发展的第一动力，力争高新技术产业增加值增长10%以上，占GDP比重不断增加。加大传统产业技术改造，突出抓好50个投资3000万元以上技改项目，鼓励劳动密集型企业实施机器换人计划。进一步实施高新技术企业成长工程，力争新增国家高新技术企业10家。强化企业创新主体地位和主导作用，坚持市场导向，深化产学研合作，加强自主创新和协同创新，引导创新资源向企业聚集，提高科技成果转化应用能力。更加积极地引进培养人才，建立健全“人才+项目+平台”的创新人才培养开发体系。加快实施品牌战略，新增中国驰名商标1件以上、省著名商标5件以上，不断提升产品知名度和竞争力。

（四）全力加快“神韵随州”建设。继续深化新一轮“五城同创”，大力推动城镇规划上水平、城镇建设上速度、城镇管理上台阶，实现城市提神增韵。

1、不断增强城市承载能力。认真谋划城市“成长坐标”，抓好布局、规划和设计，高水平编制《随州市城乡总体规划》，坚持一张蓝图画到底。围绕道路桥梁、园林绿化、节能减排、民生保障、供水供电、公共服务等方面，投资100亿元以上，推进实施一批重点市政工程，确保完成麻竹高速浪河互通、城南新区中环路、解放西路改造、汉东东路延伸段等项目，基本完成交通大道综合整治，继续实施东护城河治理，开工建设城市公共活动综合体、水一桥改造、编钟大道延长线浪河至何店段、城南污水处理厂、新中心客运站等项目。通过持续推进市政项目建设，促进城市功能不断完善、城市形象不断提升。

2、切实提高城市管理水平。围绕“智慧随州”建设，推进城市管理精细化、数字化、市场化，加快城市地下管网综合管廊和“海绵城市”建设，不断完善城市路网，提升道路、桥梁等市政设施的维修养护水平。提高城管综合执法水平，着力消除盲点、破解难点，推进市容环境专项整治，营造更加整洁有序、优美和谐的人居环境。完善物业企业管理体制，启动物业企业服务质量等级评定工作，分类整治规范物业管理。

3、努力推动“美丽乡村”建设。坚持城乡一体化发展，不断加快新农村建设步伐，科学编制历史文化名镇、传统村落保护规划，大力发展中心村、保护特色村，推进特色景观旅游名镇名村示范建设，创建一批美丽宜居村庄，打造一批地域特色鲜明的秀美村镇。深化“洁美家园大行动”，继续开展农村生活垃圾治理专项行动，着力改善农村生产生活环境。

4、加大生态环境保护力度。认真落实新《环保法》，严格环保执法，继续开展“零点行动”等专项整治活动，扎实做好水、大气、土壤等污染防治工作，积极实施生态修复，致力改善环境质量。着力开展生态文明示范建设，推进城乡绿色革命，深化“绿满随州”行动，建好随南“百里画廊”，打造“一轴一环三片”绿色格局，确保中心城区新增绿地面积60万平方米，促进人与自然和谐共生。

（五）全力提升开放开发层次。坚持把扩大开放作为带动全局的综合性战略举措，以开放促改革、促发展。

1、把招商引资作为开放开发的首要任务。进一步创新招商方式，围绕特色产业、重点产业、优势产业组建招商团队，瞄准项目，实行精准招商，推进园区招商、网上招商、以商招商。进一步拓宽招商渠道，精心组织参加鄂港粤经贸洽谈会等重大招商活动。进一步优化考核机制，激发招商引资的积极性、主动性，确保引进投资过亿元的项目40个以上。

2、把扩大出口作为开放开发的关键抓手。支持企业抢抓国家“一带一路”战略机遇，大力开展多种形式的对外投资和跨国经营，输出产能、技术和管理，积极发展服务贸易和服务外包，千方百计挖掘出口潜力，引导企业巩固传统市场，开拓新兴市场。加快出口产品升级换代，提高产品附加值，进一步扩大农产品、专用汽车、电子信息等特色产品出口份额。

（六）全力实施精准扶贫脱贫。围绕“五年任务、三年完成”奋斗目标，对照时间节点、脱贫任务等要求，逐级签订军令状，层层绘制作战图，突出“精准”二字，坚决打赢脱贫攻坚第一战。探索多渠道、多样化的扶贫路子，认真做好“开发扶持脱贫一批、助学扶智脱贫一批、低保兜底脱贫一批、医疗救助扶持一批、危房改造搬迁一批、基础设施建设助推一批”等工作,分类扶持贫困家庭，实现脱贫5万人。抓好产业扶贫，加大贫困乡村特色资源、优势资源、闲置资源开发力度，着力建设一批特色化、专业化产业扶贫基地，增强贫困地区造血功能。抓好项目扶贫，完成126个重点贫困村光伏电站建设，加快实施1743户易地扶贫搬迁，加大农村电网升级改造力度，推进危房改造、水库除险、中小河流治理、小农水建设。抓好资金扶贫，统筹用好财政扶贫资金，积极引导社会资本参与扶贫开发，加大小额扶贫贷款力度，确保贷款规模达到1亿元以上。抓好结对定点扶贫，进一步提高精准度和有效性。

（七）全力改善民生保障福祉。坚持民生导向，守住底线、完善制度、引导预期，切实保障群众基本生活和基本公共服务，促进社会公平正义，提升人民幸福指数。

1、多谋富民之策。实施更加积极的就业政策，努力创造更多就业机会，进一步健全技能培训、信息发布等就业服务体系，重点做好高校毕业生、退役军人、残疾人等就业服务工作，全面打造创业政策、融资、孵化、培训等平台，加快建设创业孵化基地，更好推进全民创业。落实强农惠农政策，发展壮大致富产业，提高农民从事种植业、养殖业、加工业和服务业的增收致富能力。

2、办好利民之事。推动学前教育、义务教育、高中教育、职业教育、特殊教育协调发展，切实改善办学条件，加强教师队伍建设，推进素质教育，提高教育质量。健全覆盖城乡的公共文化服务体系，继续推进“三馆一站”免费开放、送戏下乡、全民阅读等文化惠民工程，做好非物质文化遗产保护和传承，广泛开展群众性体育活动。全力抓好市中心医院新院、市中心血站新站建设，推动乡镇卫生院、村卫生室、社区卫生服务中心提档升级，扩大基本公共卫生服务覆盖面。做好计划生育政策宣传引导，依法组织实施全面二孩政策，切实抓好出生人口性别比治理。加大“餐桌污染”治理力度，不断推进“食品放心工程”。

3、履行安民之责。完善多层次社会保障体系，完成社会保险扩面征缴工作，实施“全民参保登记计划”，建立全民参保基础数据库，推广使用全国统一的社会保障卡，提高社会保险参保覆盖率和待遇水平。住房公积金新增归集5.5亿元，确保资金使用率达到90%以上。稳定房地产市场，积极消化库存，促进房地产业健康发展。加强和创新社会治理，充分发挥网格化管理功效，进一步提升社会治理效能和服务水平。强化社会治安综合治理，进一步健全社会矛盾大调解机制，严厉打击各类违法犯罪，深入推进“平安随州”建设。认真落实安全生产责任制，加强信息化、标准化“两化”体系建设，建立安全隐患排查整治长效机制，坚决遏制重特大事故发生。加强精神文明建设，认真践行社会主义核心价值观,继续办好“道德讲堂”，深入开展“十星创建”，牢牢把握正确舆论导向,营造良好舆论氛围。

（八）全力加强政府自身建设。新形势、新任务对政府工作提出了新的要求，必须不断增强政府的执行力和公信力，努力建设组织放心信任、人民满意信赖的政府。

1、坚持把依法行政作为基本准则。弘扬法治精神，推进依法治市，带头维护宪法和法律权威，主动接受人大法律监督、政协民主监督和社会舆论监督，将法治思维和法治方式贯穿政府工作的方方面面。坚持依法行政，完善政府法律顾问制度，严格执行行政机关负责人出庭应诉制度，推进政府决策科学化、民主化、法治化。深化行政执法体制改革，规范自由裁量权，压缩弹性执法空间，加强执法监督，实施行政执法全过程记录，确保严格规范公正文明执法。

2、坚持把为民服务作为执政根本。始终把为人民服务作为一切工作的出发点和落脚点，从人民群众最需要的地方做起，从人民群众最不满意的地方改起，努力让人民群众享受更多发展成果。进一步深化“三民”工程，加快建设幸福社区，积极打造市、县、乡、村“四级互联”的“e+”为民服务综合平台。深入开展大接访活动，完善首接负责、限期办结机制，畅通民意诉求渠道，及时回应群众关切。完善政务公开制度，推行权力清单和责任清单制度，以行政权力“瘦身”推动市场主体“强身”，让权力在阳光下运行。

3、坚持把改进作风作为坚实保障。巩固党的群众路线教育实践活动和 “三严三实”专题教育活动成果，持续抓好整改事项落实，把纪律和规矩挺在前面，防止“四风”反弹回潮。加强对公务员的教育、管理和监督，努力建设一支政治强、业务精、作风正的公务员队伍。加大督办检查力度，着力解决“不作为、慢作为”问题，形成勇于负责、敢于担当、重在落实的优良作风。健全考核奖惩机制，严格奖优罚劣、奖勤罚懒，汇聚干事创业的正能量。

4、坚持把清廉从政作为履职底线。扎实推进“清廉随州”建设，认真贯彻新修订的 《廉洁自律准则》、《纪律处分条例》，严格落实党风廉政建设 “两个责任”，始终保持惩治腐败的高压态势，依法治理和纠正损害群众利益的突出问题，真正做到“清廉为官、事业有为”。严肃财经纪律，厉行节约，反对浪费，严控“三公”经费支出，重点加强工程建设、土地管理和政府采购的行政监察和审计监督，确保公共资金使用安全，促进干部清正、政府清廉、政治清明。

各位代表，今年市政府将继续办好一批民生实事：（1）提升公共就业服务水平，切实帮助就业困难对象再就业，确保城镇新增就业2万人；努力推进大众创业，全年新增发放创业贷款1亿元，扶持创业1000人。（2）不断改善义务教育薄弱学校基本办学条件，投入1亿元新建、改扩建50所中小学校舍4万平方米，确保白云湖学校春季正式开学、胜利学校完成主体工程；筹措资金4000万元，资助3万名贫困学生。（3）加快保障性住房建设，新筹集公租房房源600套，改造城市棚户区8500户。（4）创新完善立体化社会治安防控体系，加强人防、物防、技防建设，全面组建城市社区专职辅警队，建立80个农村中心村巡防队，实现城市重点区域A类公众视频监控全覆盖。（5）进一步改善公共交通，建设推进一批公交综合服务站和公交枢纽站、首末站，建成通村公路350公里；新建停车场5个、停车位2180个；开展交通秩序专项整治，完成交通大道等城市重点路段改造，努力缓解城区交通拥堵问题。（6）推行环卫市场化改革，基本完成中心城区旱厕改造，推动城市环境状况明显改观。（7）加快便民市场建设，改造3至5个农贸市场，建设100家小餐饮示范店。（8）建成滨湖健康主题公园，完成随城山森林公园一期工程，推进漂水湿地公园建设，在中心城区再兴建一批街头小游园，再建设50个农村 （社区）文化广场。（9）持续推进城区饮用水源地综合治理，全面完成白云湖排污口封堵截流，开展城区黑臭水体治理，再实现10个乡镇集中式饮用水源地规范化建设达标。 （10）持续抓好大气污染防治工作，加大 “两禁、三控、四治”落实力度，实施中心城区全面禁鞭，力争PM10均值比上年继续下降。

各位代表，建市15年来，随州已经走过不平凡的发展历程，当前正在奋力开拓全面建成小康社会的伟大征程。让我们在市委的坚强领导下，团结和依靠全市人民，继往开来，真抓实干，砥砺前行，奋勇争先，不断开创“圣地车都”建设新局面，为谱写随州人民美好生活新篇章而努力奋斗！
